# Prevention of progression in Parkinson’s disease

**DOI:** 10.1007/s10534-018-0131-5

**Published:** 2018-07-20

**Authors:** Jan Aaseth, Petr Dusek, Per M. Roos

**Affiliations:** 1 Research Department, Innlandet Hospital Trust, Brumunddal, Norway; 2 Inland Norway University of Applied Sciences, Elverum, Norway; 3 Department of Neurology and Center of Clinical Neuroscience, First Faculty of Medicine, Charles University, Praha 2, Czech Republic; 4 General University Hospital in Prague, Prague, Czech Republic; 5 Department of Radiology, First Faculty of Medicine, Charles University, Praha 2, Czech Republic; 6 Institute of Environmental Medicine, Karolinska Institutet, Solna, Sweden; 7 Department of Clinical Physiology, Capio St. Görans Hospital, Stockholm, Sweden

**Keywords:** Substantia nigra, Dopamine, Iron, Copper, Oxidative stress, Metal binding

## Abstract

Environmental influences affecting genetically susceptible individuals seem to contribute significantly to the development of Parkinson’s disease (PD). Xenobiotic exposure including transitional metal deposition into vulnerable CNS regions appears to interact with PD genes. Such exposure together with mitochondrial dysfunction evokes a destructive cascade of biochemical events, including oxidative stress and degeneration of the sensitive dopamine (DA) production system in the basal ganglia. Recent research indicates that the substantia nigra degeneration can be decelerated by treatment with iron binding compounds such as deferiprone. Interestingly compounds known to decrease PD risk including caffeine, niacin, nicotine and salbutamol also possess iron binding properties. Adequate function of antioxidative mechanisms in the vulnerable brain cells can be restored by acetylcysteine supplementation to normalize intracellular glutathione activity. Other preventive measures to reduce deterioration of dopaminergic neurons may involve life-style changes such as intake of natural antioxidants and physical exercise. Further research is recommended to identify therapeutic targets of the proposed interventions, in particular protection of the DA biosynthesis by oxygen radical scavengers and iron binding agents.

## Introduction

**Fig. 1:**
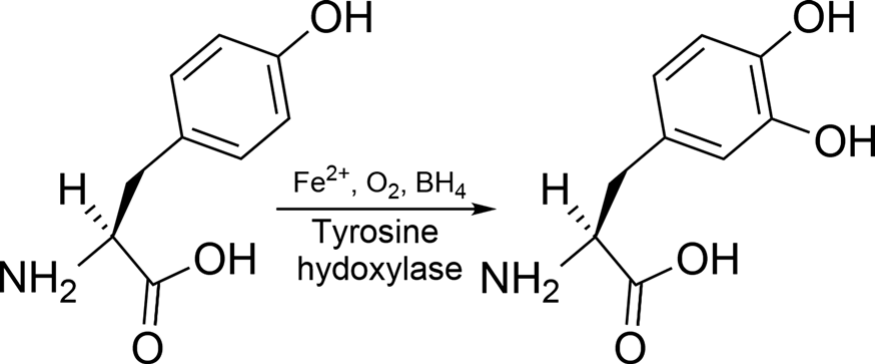
The enzyme *tyrosine hydroxylase* uses three cofactors, viz. Fe(II), molecular oxygen and tetrahydrobiopterin (BH_4_) in the biosynthesis of l-*dopa*. One of the oxygen atoms in O_2_ is used to hydroxylate the tyrosine molecule in meta-position to l-dopa, and the other is used to hydroxylate the cofactor BH_4_. The oxidation state of the iron atom is crucial. If the Fe(II) is oxidized to Fe(III), or replaced by another metal, the enzyme is inactivated. Inappropriate interactions between Fe, or other transition metals, with the enzyme or its reactants generate toxic amounts of reactive oxygen species

Parkinson’s disease (PD) affects at least 6 million people worldwide (Kalia and Lang 2016) and typically occurs in people over the age of 60, of whom about one percent are affected (Global Burden of Disease Cancer et al. 2017). Life expectancy is moderately reduced yet PD mortality doubles some 15 years after the diagnosis. PD is a progressive disorder of the brain where dopamine-producing cells located in the substantia nigra (SN) degenerate. Dopamine (DA) synthesized in SN is an important neurotransmitter depleted both in PD and other movement disorders (Bernheimer et al. 1973). The precursor of DA known as l-dopa originates from the amino acid l-tyrosine through the action of the enzyme tyrosine hydroxylase that uses oxygen and iron (Fe) as cofactors (Fig. 1). Dopamine is transported extracellularly by the DA active transporter to synaptic receptors on neurons located in the striatum, nucleus accumbens, hippocampus, neocortex, and to the spinal cord. Dopamine deficiency in the nigrostriatal pathway is the ultimate cause of the parkinsonian syndrome, i.e. presence of bradykinesia and at least one of rigidity or resting tremor (Postuma et al. 2015).

Neurodegenerative changes in PD are associated with progressive alpha-synuclein aggregation and clinical symptoms developing slowly over time. It has been shown that alpha-synuclein pathology with Lewy bodies is present in gut myenteric plexus and olfactory nerve decades before motor symptoms develop. According to prevailing theory, the progressive alpha-synuclein pathology occurs in a prion-like manner and ultimately affects the entire nervous system. Some neuronal populations appear to be more vulnerable to the alpha-synuclein pathology than other. This is the case of SN dopaminergic neurons, which are prominently affected by neurodegeneration in PD (Poewe et al. 2017). Neither the etiology of the alpha-synuclein related neurodegeneration nor the cause of the vulnerability of dopaminergic cells is currently known. It is however clear that ageing, as well as genetic and environmental factors are involved (Kalia and Lang 2016). Specific mutations (Kalinderi et al. 2016) in genes causing familial PD as well as polymorphisms increasing the risk of PD have been identified. Interestingly, mechanisms of these mutations appear to converge on disrupted synaptic, endosomal, and lysosomal trafficking ultimately leading to overwhelming of cellular disposal mechanisms (Abeliovich and Gitler 2016).

Apparently the development of PD is characterized by three different biochemical dysfunctions, viz. abnormal protein aggregation, inhibition of mitochondrial complex 1, and oxidative stress (Dauer and Przedborski 2003; Tanner et al. 1989). In normal healthy states, aggregates of alpha-synuclein are cleared by autophagy provided adequate activity of leucine-rich repeat kinase 2 (LRRK2) is present (Volpicelli-Daley et al. 2016). Mutations in the LRRK2 gene have been noted in sporadic and in familial PD (Berg et al. 2005). Mutations in other genes, i.e. the SNCA gene (the alpha-synuclein gene), also represent risk factors for PD (Kalinderi et al. 2016).

However, genetic background is considered to account for only about 10% of PD cases, suggesting that other factors play a crucial role in the pathogenesis. History of recent cranial trauma (Fang et al. 2012a) and CNS infections (Fang et al. 2012b) as well as environmental factors such as exposure to fungicides and pesticides, e.g., maneb, rotenone and paraquat (Costello et al. 2009; Tanner et al. 2011) seem to be involved in the pathogenic process. There is ongoing discussion whether chronic low-level exposure to various metals such as manganese may also be a risk factor for PD (Lucchini et al. 2009). Furthermore, extensive epidemiological research has identified several factors, such as tobacco smoking, drinking tea or coffee, use of NSAID, statins, salbutamol, and physical activity (Yang et al. 2015) to pose a reduced risk to develop PD (Ascherio and Schwarzschild 2016; Barranco Quintana et al. 2009; Noyce et al. 2012). It is an interesting question whether these factors may prevent aggregation of alpha-synuclein itself or protect vulnerable cellular populations from its toxic effects. Indeed, recent research indicates that there is no correlation between Lewy body load and dopaminergic neuronal loss in incidental Lewy body disease and PD cases (Iacono et al. 2015) suggesting that alpha-synuclein pathology does not irrevocably lead to neuronal death.

At present there is no effective cure for PD, and the current treatment is merely symptomatic based on substitution of dopaminergic deficit. Disease-modifying approaches are urgently needed and understanding the effects of protective factors identified by epidemiological studies may bring us closer to this goal.

The present review identifies common mechanism of some preventive measures in PD and highlights their potential role in ameliorating metal-related pathology. We also discuss links between metal exposure, Fe metabolism and DA metabolism.

### Dysregulated iron and copper metabolism and oxidative stress in Parkinson’s disease

The accumulation of Fe observed in SN in association with the occurrence of aggregated misfolded protein seems to contribute to the progression of PD (Ward et al. 2014). One theory claims that pathological distributions of Fe and copper (Cu) (Dusek et al. 2015; Genoud et al. 2017; Ward et al. 2014) aggravate oxidative damage and contribute to PD progression. Elevated cytosolic Fe in SN of PD patients has long been associated with neurotoxicity via various mechanisms including deleterious interactions between DA and Fe (Hare and Double 2016). Furthermore, dysmetabolism of Cu with reduced cytosolic fraction of the metal, reflecting reduced activity of the cuproenzyme superoxide dismutase-1 (SOD1) (Genoud et al. 2017) will also increase the oxidative stress in PD (Trist et al. 2018). The pathogenic role of the aggregation of alpha-synuclein, effective as a Cu chaperone, for the SOD1 insufficiency is unclear (Barnham and Bush 2008). The alterations in Fe and Cu distribution appear to occur early in the PD disease process and are therefore not considered to represent merely a reactive redistribution of metals secondary to neuroinflammation (Genoud et al. 2017).

The neurotransmitter precursor l-dopa is synthesized from the amino acid tyrosine by the enzyme complex tyrosine hydroxylase that uses molecular oxygen and Fe(II) as cofactors (Fig. 1). This enzyme complex represents a sensitive biochemical site in the SN. Transition metal ions, including free ions of Fe, manganese, and Cu, can catalyze the generation of ROS around this site, and thereby affect the enzymatic function of tyrosine hydroxylase negatively. Malfunction of tyrosine hydroxylase may further speed up ROS generation, forming a vicious circle of enzymatic dysfunction and ROS generation accelerating nigrostriatal cell death. However, the exact mechanisms of cellular death in PD, starting in the SN pars compacta and subsequently spreading to other CNS regions, are yet not fully known (Kalia and Lang 2016). Environmental pollutants contributing in the PD pathogenesis include 1-methyl-4-phenyl-1,2,3,6-tetrahydropyridine (MPTP) (Langston and Ballard 1984), paraquat, rotenone, and maneb (Liou et al. 1997; Pezzoli and Cereda 2013; Qi et al. 2014). The toxic effects of rotenone and MPTP are ascribed to their impact on the mitochondrial electron transport chain, as they cause a transport block of electrons in complex 1, leading to generation of ROS (Gao et al. 2003; Smeyne and Jackson-Lewis 2005). Paraquat is another catalyst for the formation of ROS (Bus and Gibson 1984). In rats paraquat toxicity has been associated with Parkinson-like neurodegenerations (Ossowska et al. 2006). One study reported a link between combined exposure to paraquat and Fe in infancy and mid-life Parkinson’s in laboratory mice (Peng et al. 2007). Exposure to maneb, a manganese dithiocarbamate derivative, is associated with increased deposition of transition elements in cerebral regions (Aaseth et al. 1981), thereby apparently causing dopaminergic neurodegeneration (Zhang et al. 2003).

### Reduced PD progression by deferiprone and other iron chelators

A characteristic feature of the PD neuropathology is the accumulation of Fe in the degenerating SN (Ward et al. 2014). Although neurodegenerative changes are widespread in the PD nervous system, increased Fe concentrations is apparently limited to SN (Acosta-Cabronero et al. 2017; Double et al. 2010) where the most pronounced neuronal loss is reported (Barbosa et al. 2015). Interactions between electrophilic Fe(III) ions and an extra-vesicular fraction of DA are assumed to play a critical role in the progressive cellular degeneration, initially creating an unstable Fe(III)–DA-complex, that gives rise to neurotoxic products especially in SN and related sensitive regions of the brain (Hare and Double 2016). The unstable Fe(III)–DA complex appears to bring about the production of *o*-quinones (Zhang et al. 2012) and DA-quinones (DA-quinones), as well as 6-hydroxydopamine (6-OHDA). The former compounds are basis of the pigment neuromelanin while the latter agent is a well-known neurotoxin frequently used in animal PD models. Inside the cell, unbound DA-quinones react with the sulfhydryl groups of glutathione (GSH) and protein thiols to form altered protein structures (Stokes et al. 1999) that cause cellular toxicity and microglial activation (Asanuma et al. 2003). It has been suggested that formation of 6-OHDA initiates a cascade of reactions that increases the intracellular labile Fe pool, thus overwhelming protective antioxidant mechanisms (Hare and Double 2016). Other catecholamines also form unstable complexes and toxic products with Cu(II) or Fe(III) (Aaseth et al. 1998). Interestingly, a more stable chelate is formed between Fe(III) and the ephedrine derivative, salbutamol (Fatima 2012), a commonly used anti-asthmatic drug.

Intriguingly, use of the latter drug was associated with significantly decreased risk of developing PD in a large epidemiological trial. Additional analyses have shown that the protective effect is likely based on its documented role as a regulator of the alpha-synuclein gene decreasing its expression (Mittal et al. 2017). A large-scale study is about to be launched studying salbutamol as a disease-modifying agent for PD (Robinson 2017). It would be interesting to see to what extent the chelating properties of salbutamol contribute to its putative neuroprotective effect.

**Fig. 2:**
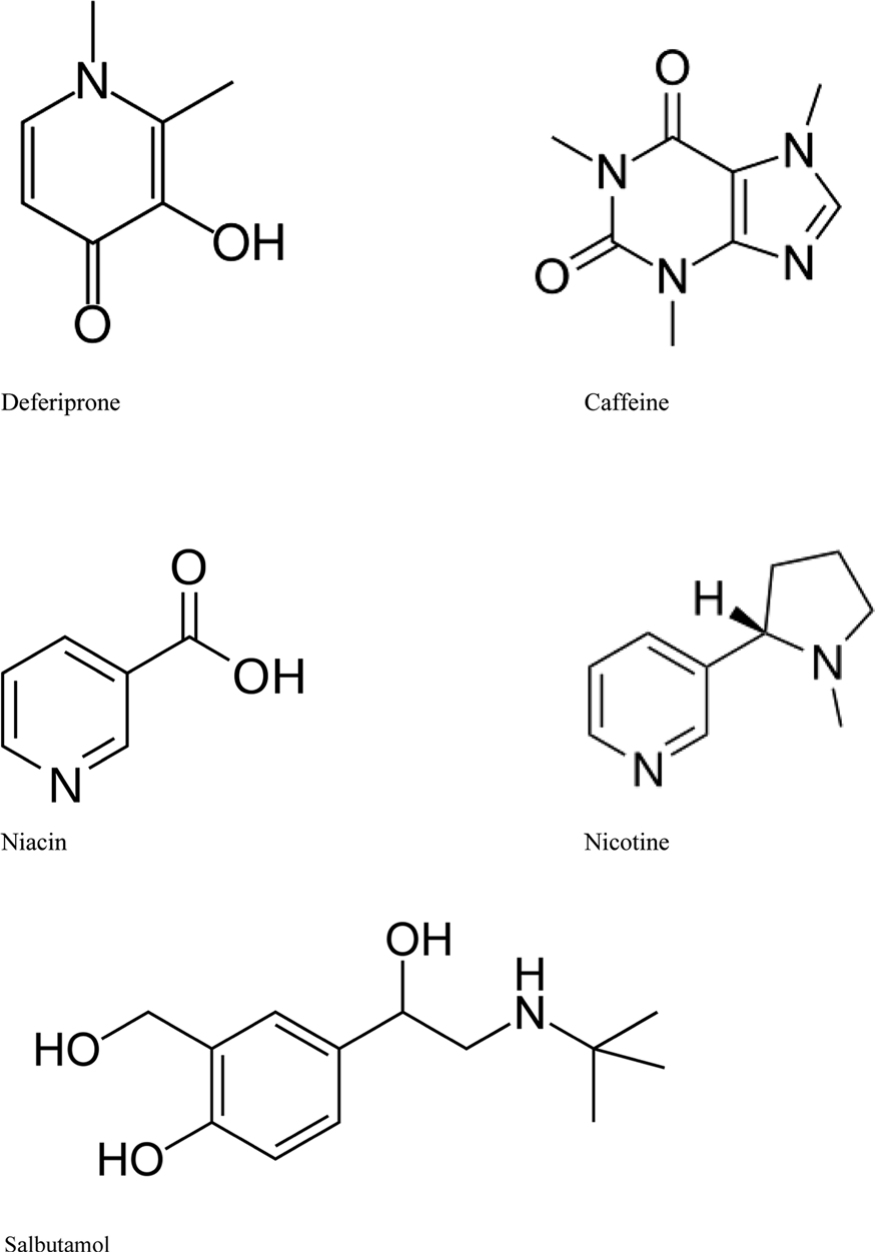
Chemical structures of proposed protective agents deferiprone, caffeine, niacin and nicotine, showing their nitrogen and oxygen electron donor groups, responsible for the affinity to electrophilic ions of transition metals, e.g. Fe(III) and Cu(II)

Another metal-binding drug, deferiprone, is a hydroxy ketone pyridine derivative (Fig. 2) effective as an Fe chelating agent in clinical settings and known to cross the blood–brain barrier (Roy et al. 2010). Devos et al. (2014) studied the effect of conservative Fe chelation with 30 mg/kg/day of deferiprone in PD patients. This double-blind placebo-controlled pilot trial showed that 12 months of deferiprone therapy decreased disease progression by three points on the Unified Parkinson’s Disease Rating Scale part III (UPDRS-III) compared to the placebo group. Additionally, quantitative R2* transverse relaxometry MRI technique, a surrogate marker of tissue Fe concentrations, confirmed that deferiprone led to a drop in SN Fe content (Devos et al. 2014). Another placebo-controlled deferiprone trial in PD patients showed only a trend for UPDRS-III improvement in the group using the dose 30 mg/kg/day, but the R2* MRI indicated a decrease of Fe in the dentate and caudate nuclei in the active group compared to placebo (Martin-Bastida et al. 2017). Statistical significance was not reached, maybe related to the short duration (6 months) of this trial.

These results support the hypothesis that conservative Fe chelation may modify natural progression of PD. It is possible that Fe chelation may be even more effective when initiated in prodromal stages of PD.

### Caffeine: maybe another iron chelator

Postuma et al. (2012) found that patients with PD improved by taking caffeine pills for 2 months. For the study, 61 PD patients with a mean age of 60 years were selected. In six weeks, 30 of the participants received caffeine pills in an amount equivalent to two cups of coffee a day and the remaining 31 subjects got placebo capsules. At the end of the study, the patients who had ingested caffeine reported, in addition to less drowsiness, a general improvement in motor symptoms such as muscle stiffness and movement difficulties (Postuma et al. 2012). A larger multicenter follow up study was however unable to reproduce these findings (Postuma et al. 2017).

Ross et al. (2000) upon analyzing data collected during 30 years of follow-up of 8004 Japanese-American men aged 45–68 enrolled in the study during the period 1965–1968, identified 102 men with PD, and noted that high coffee or caffeine intake was linked to a significantly lower frequency of PD. This effect appeared to be independent of smoking. Their data indicated that the mechanism was related to the caffeine dose and not to other nutrients (Ross et al. 2000). Animal studies (Xu et al. 2016) and meta-analysis (Costa et al. 2010) have confirmed these observations. Another meta-analysis has shown an inverse linear dose–response relationship between PD risk and amount of coffee, tea or caffeine consumed. It was found that maximum protection was reached at about three cups of coffee per day (Qi and Li 2014). In this context the structural similarities between deferiprone and caffeine should be noted (Fig. 2). Protective effects of caffeine may be related to its ability to bind Fe via its nitrogen and oxygen groups, albeit with a lower binding constant than the EDTA-Fe complexes (Andjelković et al. 2006).

### Niacin is a protector with metal binding properties

Vitamin B3, or niacin, also known as nicotinic acid, may alleviate certain types of early-onset PD symptoms (Anderson et al. 2008). Niacin has been shown to attenuate neuroinflammation through an action on niacin receptor 1 (NIACR1), also known as GPR109A and may have a therapeutic potential toward PD (Wakade and Chong 2014). Although moderate amounts of niacin are found in a number of foods, including chicken, turkey, beef, peanut and mushrooms, the vitamin can be supplemented in therapeutic doses as tablets. In MPTP exposed rodents, the administration of nicotinamide gave a dose-dependent saving of striatal DA levels and SN neurons (Anderson et al. 2008). Niacin, which is a precursor for nicotinamide adenine dinucleotide (NAD–NADH) needed for DA production, may serve several purposes, i.e., reduce inflammation through NIARC1-related mechanisms, increase DA synthesis in the striatum through NADPH supply and increase NAD/NADH ratio to restore complex 1 functions in mitochondria. Niacin can also bind transition metal ions including Fe into stable complexes (Al-Saif and Refat 2012).

### Nicotine and its neuroprotective mechanisms

Nicotine may have a potential to protect against PD, and pharmaceuticals that target nicotine receptors have been searched for. In particular, the nicotinic alpha-7 receptor, implicated in long-term memory function, has been in the focus of interest (Rang et al. 2003).

Nicotine acts as an agonist to most nicotinic acetylcholine receptors (Malenka et al. 2009), and can be used to improve cognition and alertness (Jasinska et al. 2014). A meta-analysis of 41 placebo-controlled studies concluded that nicotine had a positive effect on motor abilities, orienting attention, and working memory (Heishman et al. 2010).

Using rat embryo tissue, Toulorge et al. (2011) prepared brain cell cultures demonstrating conditions that favored progressive loss of dopaminergic neurons, which also showed distinctive features otherwise characterizing PD, and this group also reported a protective effect of nicotine. In normal mice, nicotine has been found potentially able to rescue dopaminergic neurons, but apparently not in mice without the nicotine receptor (Toulorge et al. 2011).

Although one of the developed nicotine receptor agonists, varenicline, showed only limited protective effect (Bohnen et al. 2009), further research on nicotine receptors and nicotine agonists in PD brings possibilities for early stage neuroprotective treatment (Barreto et al. 2014; Kelton et al. 2000; Quik 2004). Interestingly, nicotine also acts as a metal-chelating agent, with high affinity for Fe(III) (Fazary 2017). Thus, it is tempting here to forward the hypothesis that the PD protecting potential of nicotine is not merely related to its effects on the receptors, but also to its ability to pass the blood–brain barrier better than other chelators and thus act as an intra-neuronal Fe chelating agent (Zhang et al. 2006).

### Glutathione and glutathione peroxidase protect against neurodegeneration

Another approach to ameliorate cellular deterioration caused by ROS in PD is to raise the intracellular levels of the tripeptide glutathione (GSH). Antioxidant defenses in SN are relatively low, compared to other regions of CNS, due to low levels of GSH, particularly during the early stages of PD when extravesicular DA and its degradation products may act as a GSH depleting agents (Pearce et al. 1997; Stokes et al. 1999). *N*-acetylcysteine (NAC) shows antioxidant properties by restoring cellular GSH, which participate in important endogenous antioxidant systems. In experimental studies NAC has been reported to protect against PD development (Rahimmi et al. 2015).

Glutathione acts either alone or together with an appropriate enzyme system, viz. glutathione peroxidases (GPXs), to reduce ROS. Also, GSH detoxifies xenobiotics and maintains sulfhydryl proteins in a reduced state (Meister and Anderson 1983). The antioxidant characteristics of GSH have been demonstrated in several models of oxidative stress, including models using buthionine-sulfoximine (BSO) to deplete GSH (Wullner et al. 1996). In these studies, the GSH depletion increased oxidative stress in whole cells as well as in mitochondrial fractions. Depletion of GSH with BSO potentiated the MPTP-induced tyrosine hydroxylase-positive neuron death in pars compacta of SN (Wullner et al. 1996). Furthermore, NAC treatment after MPTP or rotenone exposure in the GSH-depleted models, restored mitochondrial complex 1 and protected against DA loss in SN (Chinta et al. 2006).

It is tempting to suggest that supplementation with NAC in adequate doses to patients with PD may inhibit disease progression.

In vivo, GSH exerts most of its anti-oxidative functions as a cofactor to the GPX family of enzymes. GPXs are a group of selenium-containing enzymes capable of reducing toxic peroxides (Rotruck et al. 1973); GPX1 is present in both neurons and glial cells (Power and Blumbergs 2009). Overexpression of GPX decreases the amount of neuron loss in neurotoxic conditions (Wang et al. 2003). An immunocytochemical study of GPX1 expression showed that dopaminergic neurons in the SN expressed low levels of this protein, while other regions not affected in PD, expressed higher levels (Trepanier et al. 1996). In an experimental model of GPX1-deficient PD-mice challenged with MPTP, DA levels in vulnerable regions decreased (Klivenyi et al. 2000). However, in cortical samples taken from PD patients, GPX3 and GPX4 proteins were elevated compared to control subjects (Blackinton et al. 2009), presumably reflecting a protective response.

A marginal or deficient selenium status as is reported from Scandinavian regions (Alehagen et al. 2016; Ellingsen et al. 2009) and other parts of Europe involve a deficient GPX-dependent protection (Alehagen et al. 2017), also with regard to nigrostriatal functions. To the knowledge of the authors, clinical studies with selenium supplementation in early stages of PD in these regions have yet not been carried out.

### Non-steroidal anti-inflammatory drugs

Neuroinflammation contributes to degeneration of the dopaminergic nigrostriatal pathway in PD (Vivekanantham et al. 2015) through mechanisms of microglial activation. Pro-inflammatory phenotype of microglia is promoted by extracellular alpha-synuclein aggregates and neuromelanin complexes released from dying neurons (McGeer et al. 1988). Neuromelanin contains high concentration of various metals, namely Fe, Cu and Mn that may themselves contribute to inflammation (Tansey and Goldberg 2010). Neuroinflammation in PD is rather widespread as increased inflammatory markers, such as TNFα, IFNγ, IL-6, IL-1β, and other cytokines are consistently detected in serum and cerebrospinal fluid of PD patients (Brodacki et al. 2008). Neuroinflammation is also an early event in neuronal degeneration since microglial activation has been documented by PET in patients with isolated REM sleep behavioral disorder, considered a prodromal synucleinopathy (Stokholm et al. 2017). Given that several genes causing PD, namely *LRRK2*, *GBA*, and *SNCA*, are expressed predominantly in immune cells and/or are involved in regulating inflammatory response, the emerging hypothesis is that inflammatory dysregulation is a primary trigger of neurodegeneration (Dzamko et al. 2015). However, it is not clear to what extent is the activation of immune cells is deleterious with respect to disease progression. On one side, microglial phagocytosis may clear abnormal protein aggregates, on the other side, neurotoxic effects of chronic inflammatory reaction may accelerate neuronal loss (Deleidi and Gasser 2013).

According to epidemiological data, non-steroidal anti-inflammatory drugs (NSAIDs) can reduce the risk of developing PD (Asanuma et al. 2004; Chen et al. 2003; Wahner et al. 2007). These results are apparently in line with immunocytotoxic theories of neurodegeneration, and a neuroprotective effect of NSAID was initially ascribed to cyclooxygenase type-2 inhibition (Teismann et al. 2003). However, other studies did not confirm reduced PD risk in the NSAID users compared to controls (Becker et al. 2011; Etminan and Suissa 2006). Later studies detailed the effect of NSAID, and Wahner et al. (2007) reported that reduced risk of getting PD in NSAID users was seen only among regular users and the effect was particularly strong (OR 0.44) in people who regularly used non-aspirin NSAIDs (Wahner et al. 2007). A meta-analysis by Gagne and Power (2010) revealed only a moderately reduced risk of about 15% in non-aspirin NSAID users while no protective effect was observed in aspirin users (Gagne and Power 2010). More specifically, only regular use of ibuprofen was associated with significant reduction of PD risk (pooled OR 0.73) in a study and meta-analysis of several NSAIDs (Gao et al. 2003). Taken together, the fact that ibuprofen use, but not use of other NSAIDs, was associated with lower PD risk suggests mechanisms other than a generic anti-inflammatory activity. It is possible that only some few NSAIDs act protective, owing to their ability to form Cu, manganese or Fe chelates (Duncan and White 2012). Interestingly, it was suggested that ibuprofen possesses Fe chelating (Kennedy et al. 1990) or hydroxyl scavenging (Hamburger and McCay 1990) properties. Of interest is also that some Cu-chelates of NSAIDs appear to show SOD-mimetic activity (Roberts and Robinson 1985).

### Exercise

Parkinson’s disease can be delayed, or its consequences attenuated through regular safe physical activity. Recent scientific studies suggest a combination of aerobic activities, strength training from moderate to high intensity, as well as balance training, gait training, functional activities and exercises that require agility (Fisher et al. 2008; Goodwin et al. 2008). Experimental studies indicate that the inflammatory response is reduced, insulin sensitivity improved and damage to dopaminergic neurons reduced when exercise is combined with intermittent fasting (Mattson 2014).

Physiotherapeutic treatment should be started as soon as the diagnosis is confirmed and is a good way to help stimulate movement and quality of life as it improves the strength, coordination, and amplitude of the movements, i.e., by reducing the otherwise inexorable imbalance characterizing the progressive disease, thereby preventing contractures and falls (Ahlskog 2011).

## Conclusion

Environmental factors including exposure to metals, pesticides and drugs account for the majority of cases of Parkinson´s disease. Excessive oxidative stress accentuated by iron accumulation and abnormal protein aggregation in the nigrostriatal region of the brain are primary events in the development of the disease that need to be addressed by preventive measures. Iron is a necessary cofactor for the enzyme tyrosine hydroxylase responsible for DA synthesis, and accumulation of Fe in the SN deteriorates the function of tyrosine hydroxylase. Metal binding substances e.g. Deferiprone mobilize Fe from the SN possibly providing prevention. Caffeine, niacin, nicotine and salbutamol also seem to protect against progression by similar mechanisms. Supplementation with NAC together with selenium for optimal glutathione synthesis is recommended to reduce oxidative damage to sensitive SN cells.
